# Charging toward sustainability: MgCl_2_ doped chitosan–dextran polyblend electrolytes for energy storage device applications†

**DOI:** 10.1039/d4ra06365a

**Published:** 2024-11-19

**Authors:** Pradeep Nayak, Ismayil, Y. N. Sudhakar, Supriya K. Shetty

**Affiliations:** a Department of Physics, Manipal Institute of Technology, Manipal Academy of Higher Education Manipal 576104 Karnataka India ismayil.mit@manipal.edu ismayil.486@gmail.com +91 98454 97546; b Department of Chemistry, Manipal Institute of Technology, Manipal Academy of Higher Education Manipal 576104 Karnataka India

## Abstract

In this study, chitosan (CS), dextran (DN), and magnesium chloride (MgCl_2_) salt-based soild blend polymer electrolyte (SBPE) membranes that conduct Mg^2+^ ions have been developed. XRD revealed changes in the microstructure by incorporating MgCl_2_ salt into the polymer host due to polyblend–ion interaction, verified by Fourier transform infrared spectroscopy. At room temperature, the electrolyte membrane containing 30 wt% MgCl_2_ reached an ionic conductivity of 1.79 × 10^−5^ S cm^−1^. An ion transference number (*t*_ion_) of about 0.73 was achieved for 30 wt% of magnesium salt, suggesting the dominance of ion conduction in the SBPE sample. The SBPE membrane with the highest ion conductivity demonstrated an electrochemical stability window (ESW) of 2.66 V. Thermal degradation of the SBPE is influenced by the amount of salt incorporated in the poly-blend matrix as per TGA analysis. The discharge behaviour patterns of magnesium ion cells using the Mg|(CS + DN + MgCl_2_)|cathode setup were investigated with two distinct cathode materials. Moreover, the fabricated electrochemical double-layer capacitor (EDLC) showed non-faradaic behaviour in cyclic voltammetry (CV) studies with the specific capacitance of 36 F g^−1^ at 5 mV s^−1^. An environmentally friendly, biodegradable, and economically viable electrolyte that can effectively serve as a separator and electrolyte in devices such as magnesium-ion batteries and EDLCs has been investigated in this work.

## Abstract 2

This study introduces a biodegradable SBPE made from dextran, chitosan, and MgCl_2_, suitable for Mg-ion batteries and EDLCs. It exhibits excellent ionic conductivity with XRD, and dielectric analysis highlighting ion-polyblend interactions.

## Graphical abstract

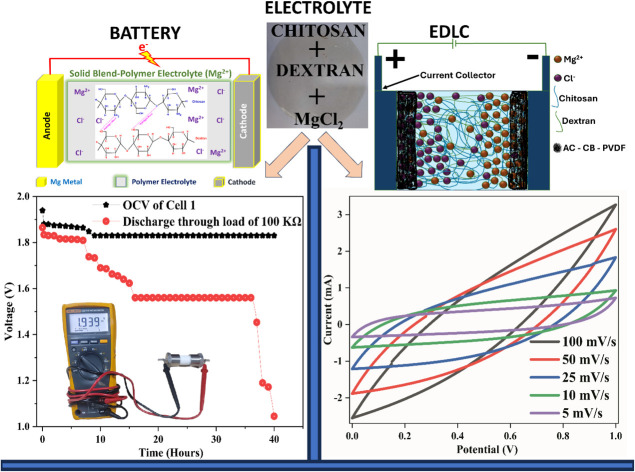

## 1. Introduction

The rising use of electronic devices has resulted in the escalating issue of electronic waste. Researchers are developing biodegradable materials to reduce this waste and replace non-biodegradable electronic components. This is an active area of research for the present scenario, and thus diverse biodegradable materials are being explored. Distinct attributes of biodegradable polymers, including their widespread availability, non-harmful characteristics, straightforward processing, and cost-effectiveness have prompted researchers to diverge from traditional synthetic to biodegradable polymers.^1^ Chitosan (CS),^2^ pectin,^3^ dextran (DN),^4^ and gellan gum^5^ are some of the most common biopolymers used to prepare solid polymer electrolytes for electrochemical devices.^6^ Liquid electrolytes have been a significant research focus for energy storage applications for three to four decades because of their high ionic conductivity. However, in recent years, there has been a shift from liquid electrolytes to solid electrolytes due to their leak-free and more compact characteristics.^7^ Solid polymer electrolytes based on biopolymers have been extensively used in batteries,^8^ electrical double-layer capacitors (EDLC),^9^ supercapacitors,^10^ photovoltaic cells,^11^ and dye-sensitized solar cells (DSSC)^12^ in the making of eco-friendly devices to mitigate their environmental impact.

Incorporating various inorganic salts into polar polymers can alter the physical and chemical characteristics of solid polymer electrolytes that in turn decide the performance of energy storage systems that involve this SBPE. Hence, selecting a suitable combination of salt and polymer host is essential to attain the desired properties for electrolytes for its applications. Several approaches have been commissioned to achieve the desirable ionic conductivity at ambient temperature, including the use of additives,^13^ polymer blending, and copolymerization.^14,15^ Polymer blending is extensively employed to create novel polymeric materials due to its straightforward preparation, cost-effectiveness, and ability to quickly adjust material properties by altering the composition.^16^ In the present work, CS and DN are employed to prepare polyblend. CS is commonly utilized as a polymer matrix in polymer electrolytes, facilitating ion dissociation due to its functional groups, such as amine (–NH_2_) and hydroxyl (–OH). Additionally, it enhances the film's stiffness.^17^ Dextran (DN) is an abundant and naturally occurring polymer;^18^ moreover, it is biodegradable, forms physical gels through non-covalent interactions, is non-toxic, and is renewable.^19^

**Fig. 1:**
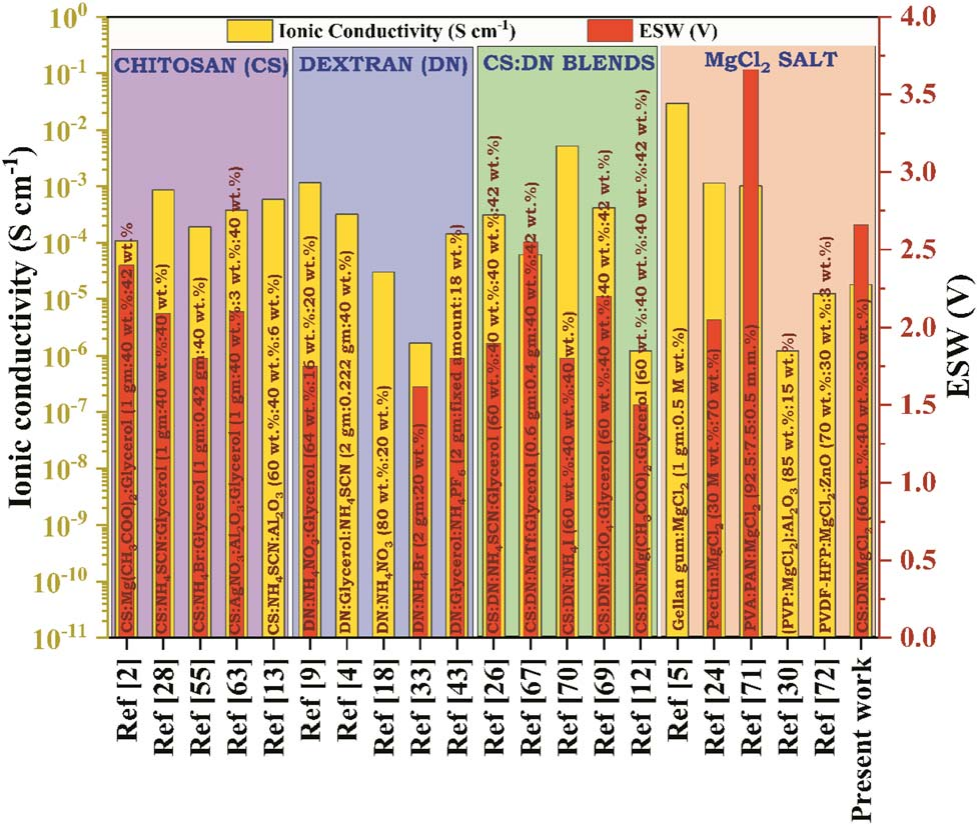
Previously documented research studies on chitosan, dextran, CS : DN SBPE, and MgCl_2_ salt, including the optimal sample's room temperature ionic conductivity and ESW values.

In a study by Aziz *et al.*,^20^ NH_4_SCN salt was employed to prepare polymer electrolyte films using DN as the polymer matrix and obtained an ionic conductivity of 3.18 × 10^−4^ S cm^−1^. The ionic conductivity and cation transference number of SBPE depends mainly on the interaction between the functional group of the DN polymer, such as O–H and C–O–C, with the salts' thus influencing charge carriers' mobility. M. F. Z. Kadir^21^ prepared a blended polymer electrolyte film of CS : DN by employing ammonium thiocyanide as the salt. The resulting ionic conductivity value was reported to be 1.28 × 10^−4^ S cm^−1^. In a study by Abdulwahid *et al.*,^22^ a polyblend of CS : DN was combined with magnesium acetate as the salt. The study yielded an observed ionic conductivity value of 1.22 × 10^−6^ S cm^−1^. They claimed that CS : DN blend film possessed less crystallinity than their counterparts. In research conducted by Aziz *et al.*,^23^ NH_4_PF_6_ salt was utilized to prepare an SBPE film consisting of CS : DN, leading to an observed ionic conductivity of 3.06 × 10^−4^ S cm^−1^. Also, an electrolyte should have at least a decomposition voltage of 1 V, and they got an electrochemical stability window (ESW) value of 1.5 V. Fig. 1 illustrates the highest conducting SBPE films derived from various polymers, such as chitosan, dextran, CS : DN polyblend, and other polymers, incorporating magnesium chloride (MgCl_2_) as a dopant, along with their respective ionic conductivity at room temperature and ESW. Limited research has been conducted on CS : DN solid blend polymer incorporating Mg salts, and there has been no report on magnesium ion conducting SBPE based on CS : DN polyblend doped with MgCl_2_ salt for its application in energy storage systems. Opting for magnesium salts offers the advantage of their abundance, manageable handling, and absence of dendrite growth, setting them apart from other options.^24^

Therefore, the current study is focused on developing and characterizing SBPE composed of chitosan and dextran. The widespread availability, water solubility, compatibility with various solvents, and remarkable ability to form films have garnered significant interest from researchers worldwide concerning DN and CS. These materials, derived from bacteria and chitin, respectively, have captured the attention of numerous scientists.^25^ The magnesium ion conducting SBPE was prepared using a host polymer matrix that included 40 wt% of DN and 60 wt% of CS.^26^ In this investigation, varying quantities of magnesium chloride were incorporated into the CS : DN blend matrix to assess the functionality of the electrolytes. Examining electrochemical characteristics aims to provide insights into the nature and process of charge transfer within these SBPEs. A highly conductive electrolyte is employed in constructing EDLCs and primary batteries.

## 2. Materials and methods

### 2.1 Synthesis of solid blend polymer electrolytes

CS, DN, and MgCl_2_ were procured from Loba Chemie Pvt Ltd. Glacial acetic acid from Emplura and deionized water were utilized as the solvents in the production of SBPE. The solution casting method produced the SBPE composed of CS : DN : MgCl_2_. Our earlier study applied the technique to formulate the SBPE samples.^27^ Briefly, DN and CS were dissolved in a 1% acetic acid solution and stirred with a magnetic stirrer for several hours until a uniform, homogeneous solution was obtained. This solution was poured onto a glass Petri dish and dried in an oven at 60 °C for 3–4 days. The resulting SBPE membranes were subsequently removed from the Petri dishes and air-dried at room temperature in a silica gel vacuum desiccator. The resulting films exhibited thicknesses spanning from 173 to 248 μm. The distribution of weight percentages for the salt and polymer components employed in this work is presented in Table 1.

**Table 1:** Composition of the solid blend polymer electrolyte samples

Sample designation	CS (g)	DN (g)	MgCl_2_ (g)
CDC0	1.20	0.80	—
CDC5	1.14	0.76	0.10
CDC10	1.08	0.72	0.20
CDC15	1.02	0.68	0.30
CDC20	0.96	0.64	0.40
CDC25	0.90	0.60	0.50
CDC30	0.84	0.56	0.60

A primary electrochemical cell consisting of an Mg(anode)|(CS(60 wt%)–DN(40 wt%)–MgCl_2_(30 wt%))|(cathode) configuration was assembled, employing the most conductive SBPE film, and the cell's performance was examined. A magnesium metal piece with a 13 mm diameter was utilized for anode preparation. In terms of cathode preparation, a combination of MnO_2_, graphite (C), and CDC30 SBPE was mixed to produce a pellet with a 13 mm diameter (used as cathode in used as cathode cell 1). Another pellet (cathode in cell 2) was created by mixing iodine (I_2_), C, and CDC30 SBPE, which was then compressed under a 5-ton pressure. Both pellets were employed to assess the performance of the primary battery.

The construction of the electrochemical double-layer capacitor involves four sequential steps. Firstly, a dry mixing process combines 81.25% activated carbon (AC) and 6.25% carbon black (CB) for 20 minutes. Secondly, the AC–CB powder is dispersed in a solution of 15 mL *N*-methyl pyrrolidone (NMP) with a 12.5% poly(vinylidene fluoride) (PVDF) binder until a thick black solution forms. Next, the solution is coated onto a stainless-steel foil using a doctor's blade. Finally, the coated foils are dried in an oven at 60 °C for one day. After drying, the electrodes are cut into squares with a geometric area of 1.5 cm^2^ and stored in a desiccator before characterization. The EDLC design configuration is AC|best-conducting SBPE sample|AC.

### 2.2 Characterizations

X-ray diffraction analysis of the SBPE samples was conducted using the Rigaku Miniflex 600 (5^th^ generation) equipment. The study covered grazing angles ranging from 5° to 80°, with a step size of 0.02°. X-ray diffraction analyses utilized monochromatic Cu K-alpha radiation with a wavelength of 1.54 Å.

Infrared spectral profiles of SBPE were obtained using a Shimadzu IR Spirit ATR-FTIR spectrophotometer, which directed infrared beams onto the SBPE at room temperature. The spectra were collected in transmittance mode with a resolution of 4 cm^−1^, covering a wavenumber range of 400 to 4000 cm^−1^.

Electrochemical impedance spectroscopy (EIS) is a valuable method for understanding the electrical properties of the SBPE.^28^ Before impedance analysis, the SBPE samples were trimmed into small pieces with a contact area of 1.13 cm^2^ and placed between the stainless-steel blocking electrodes. Impedance analysis was conducted at room temperature using a Hioki IM 3570 Precision Impedance Analyzer. The study spanned a frequency range of 100 Hz to 5 MHz, using an alternating current (AC) voltage of 100 mV. The EIS spectrum analysis software was employed to determine the prepared films' bulk resistance (*R*_b_).^29^ To assess the ionic conductivity of the electrolyte films at room temperature, eqn (1) was employed,^30^1
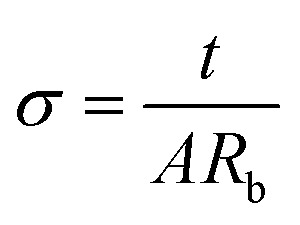


In this context, the thickness of the prepared film is represented by ‘*t*’ and the surface area of contact between the SBPE and the electrode is indicated as ‘*A*’.^31^

A FESEM (CARL ZEISS EVO 18) at 5k× magnification was used for surface morphology study. To mitigate the effects of sample charging, the specimen was coated with a thin layer of gold using a Quorum Gold sputtering device for 5 minutes. Additionally, elemental mapping was performed to evaluate the dispersion of various elements within the polymer matrix.

The thermal stability of SBPE was assessed through thermogravimetric analysis utilizing the Hitachi STA7200 TGA-DTA instrument. Approximately 2–8 mg samples were placed into a platinum crucible and heated at 10 °C per minute. Thermo gravimetric analysis (TGA) was performed from room temperature to 500 °C in a nitrogen (N_2_) gas atmosphere flowing at 20 mL per minute.

The electrical properties of the SBPE were assessed using the 2636B Keithley source meter unit to obtain data such as the electrochemical stability of the SBPE. This data was subsequently utilized to evaluate the stability of the SBPE under various electrochemical circumstances. Direct current (DC) polarization was used to measure the total ion transference number (*t*_ion_) at room temperature using eqn (2) ^32^ and electronic transference number (*t*_elec_) using eqn (3).^33^ The method involves monitoring the polarization current over time while applying a direct current at voltage of 0.5 V across the sample positioned between stainless steel (SS) electrodes. Fig. S1† illustrates the arrangement of the cell for voltage stability and transference number assessment.2
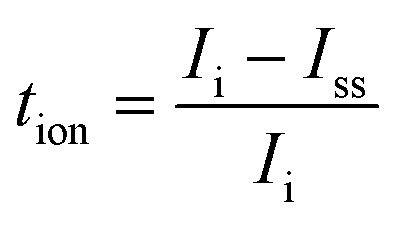

3 *t*_elec_ = 1 − *t*_ion_

where *I*_i_ is the initial current, and *I*_ss_ is the steady-state current.

A cyclic voltammogram was produced using a Biologic potentiostat (model SP50e) by sandwiching CDC30 films between Mg electrodes for one configuration and between SS electrodes for another, each at a scan rate of 100 mV s^−1^.

The initial evaluation of the assembled EDLC was performed at room temperature using cyclic voltammetry to understand the energy storage mechanism. The Biologic potentiostat SP50e was utilized with varying scan rates, spanning from 5 to 100 mV s^−1^.

## 3. Results and discussion

### 3.1 FTIR analysis

**Fig. 2:**
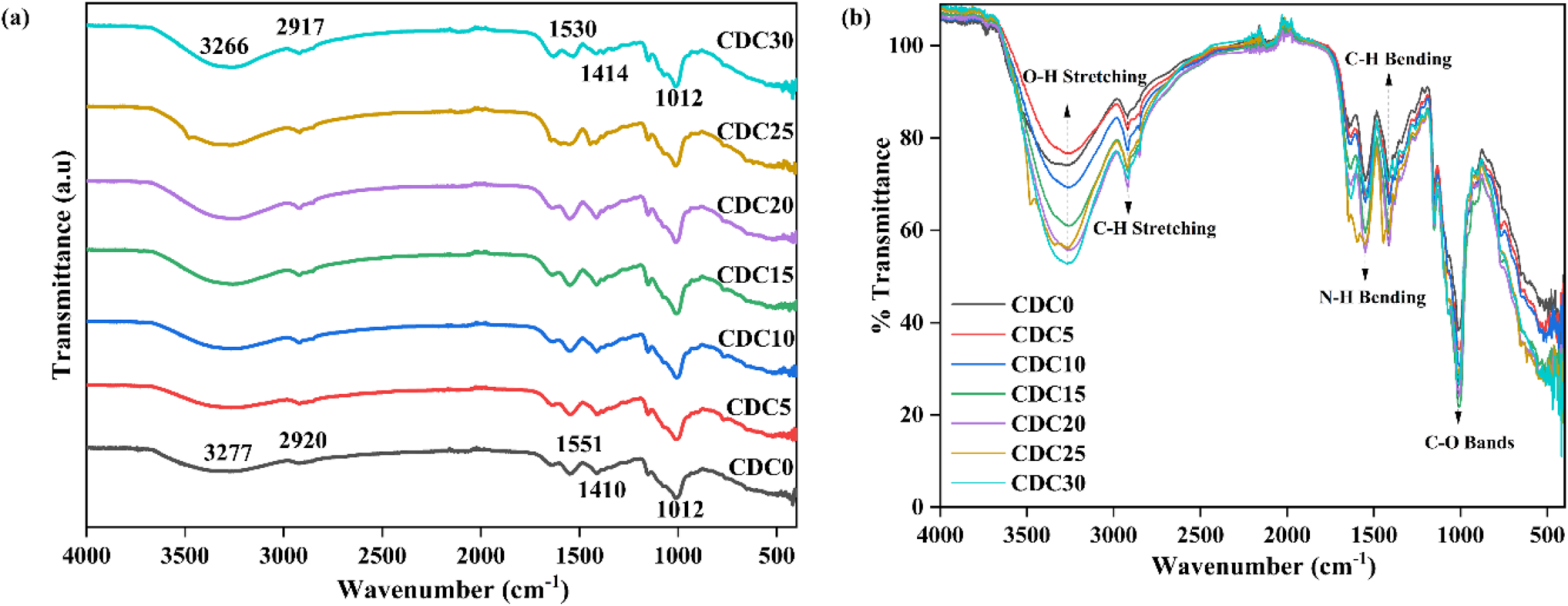
FTIR spectra of polyblend electrolyte film incorporated with a different weight percentage of the salt showing (a) wavenumber variation and (b) intensity variation.

Fig. 2(a) and (b) display the FTIR spectra of the SBPEs. These spectra offer a means to distinguish the functional groups inherent to the biopolymer. Furthermore, they shed light on the intricate interactions among the electrolyte structure's diverse components. Variations in the FTIR spectra due to polymer–polymer and polymer–ion interaction are observed either by shift, widening, and the emergence or vanishing of peaks, which can serve as indicators for recognizing the complexes formed between the solid biopolymer blend and the ions.^34^ One of the advantages of polymer blending is that it provides additional favourable complexation sites for ions to hop, thus enhancing the ionic conductivity. Complexation sites loosely bind ions and play a role in the ion conduction mechanism within the polymer electrolyte.^35^ The peak detected at around 2900 cm^−1^ corresponds to the stretching of C–H bonds within the CDC0–polyblend system.^36,37^ N–H bending modes are responsible for the peak between 1500 and 1600 cm^−1^.^38^Fig. 2(a) illustrates that incorporating magnesium chloride salt into the CS : DN blend system has altered the peak centred at 3277 cm^−1^, resulting from –OH stretching, to shift towards lower wavenumbers. This alteration signifies the physical interactions between the magnesium ions and the biopolymer blend.^39^ The C–O band exhibits a peak located approximately at 1012 cm^−1^.^40^ Table 1S† lists the different stretching and bending vibration modes. The bands of –OH stretching and –NH bending vibrations show a change of the peak towards the lower wavenumber with increased salt concentration, which strongly suggests the interaction between the CS : DN blend and the magnesium ions.^41^ As the amount of MgCl_2_ increases, the intensity of the peaks corresponding to various bending and stretching vibrations is enhanced, as shown in Fig. 2(b). All these changes indicate the interaction of the salt with the polymer blend CS : DN.

**Fig. 3:**
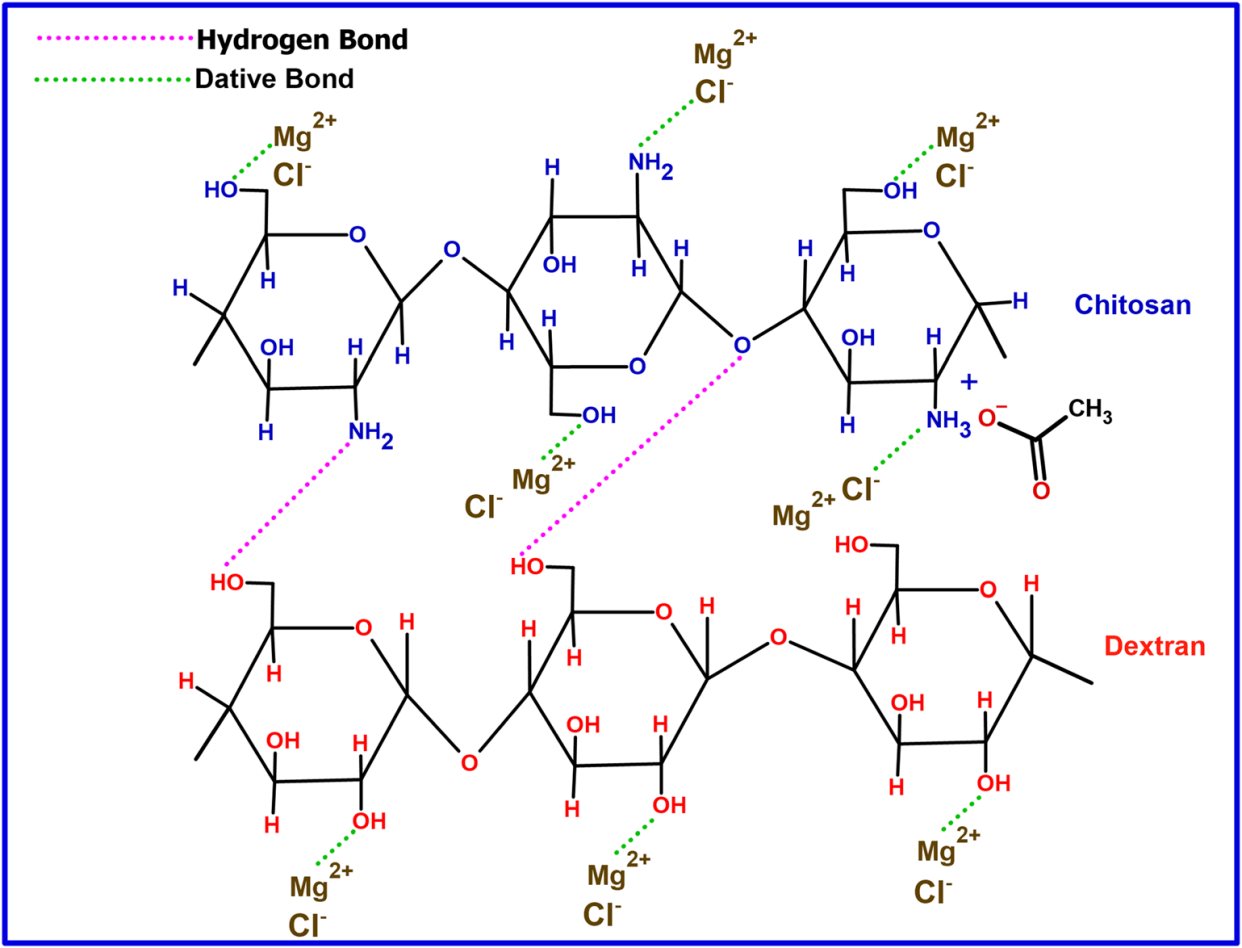
Possible interaction scheme between CS : DN polymer blend and with MgCl_2_ salt.

Fig. 3 illustrates a potential interaction between the MgCl_2_ salt and the polymer blend containing CS : DN. In this interaction, Mg^2+^ functions as a Lewis acid through electron pair acceptance, whereas Cl^−^ acts as a Lewis base by donating an electron pair. The ion–dipole complex involving Mg^2+^ and OH is confirmed by noting a shift in the wavenumber of the –OH band.^42^ A Lewis acid–base adduct results from forming a coordinate covalent bond between a Lewis acid and a Lewis base.^43^

### 3.2 X-ray diffraction (XRD)

**Fig. 4:**
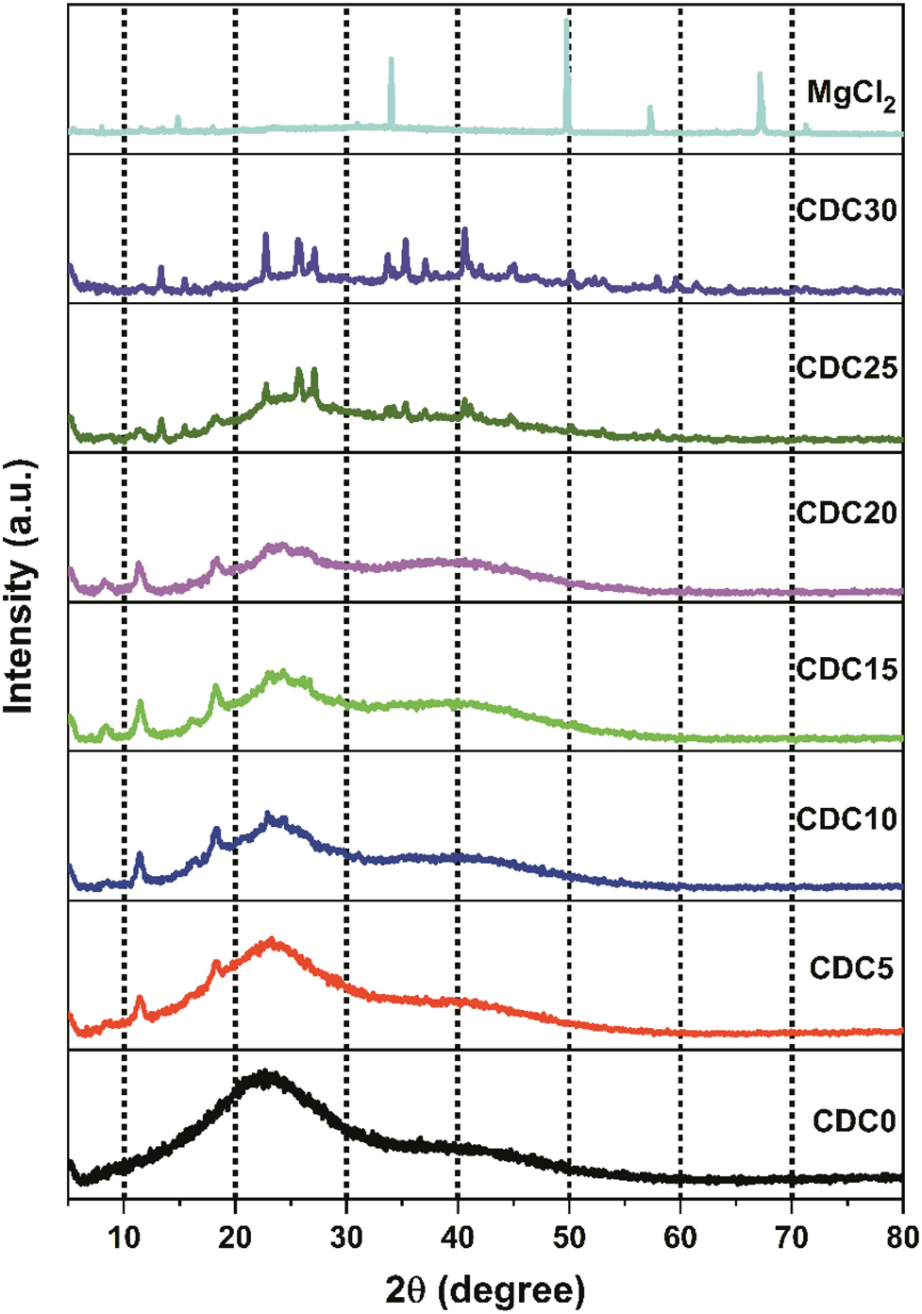
X-ray diffractogram of solid polyblend electrolyte films along with magnesium chloride salt.

Fig. 4 shows the XRD pattern of MgCl_2_ salt and salt-doped polymer electrolyte films. Due to hydrogen bonding between the hydroxyl groups of different monomers and polymer chains, the CS polymer exhibited XRD peaks at 2*θ* values of 11.3°, 18.3°, and 22.8°.^44^ The X-ray diffraction profile of DN exhibits two distinct peaks at 2*θ* angles of 17° and 22.6°, which can be attributed to the presence of intra- or intermolecular hydrogen bonding in the structure. The MgCl_2_ salt exhibits peaks at 2*θ* angles of 14.9° ((0 0 3) plane), 33.8° ((0 0 4) plane), 50° ((1 1 0) plane), 57.3°, and 67°.

The diffractogram shows that CDC0 has a single curved peak at approximately 21°. This broad peak at around 21° suggests that the CS : DN polyblend is composed of a non-crystalline structure. Blending is crucial in decreasing crystalline properties, leading to an enhancement in conductivity.^43^ As reported by O. G. H. Abdullah *et al.*^45^ incorporating salt into the polymer electrolyte disturbs the polymer's crystalline structure, facilitating ion movement. This attribute enhances ion transport speed and improves the performance of electrochemical devices. The widening of peaks and reduction in peak intensity observed in the XRD spectra indicate a decline in the crystallinity of the polymer electrolyte. Ions can diffuse through the polymer electrolyte more quickly because a less ordered crystalline structure lowers the energy barrier for ion conduction.^15,46,47^

The complexes formed between the polar groups of the biopolymer and the Mg^2+^ ions from the salt disturb the hydrogen bonding among the polymer chains. Consequently, this disturbance prevents the polymer chains from organizing into a crystalline structure. This results in a decrease in the crystallinity of the SBPE.^48^ There were some peaks in the diffractogram of CDC25 and CDC30, which are not because of the magnesium salt; these peaks are absent in the XRD of the salt. The salt interacts with any one polymer, leading to a salting-out effect. So, the blend breaks, and salt individual polymer interaction increases, leading to visible crystalline structures. The prominent peak observed around 11.3°, characteristic of CS, is consistently present in the samples ranging from CDC5 to CDC20. However, this peak is absent in the CDC25 and CDC30 samples, suggesting that magnesium chloride salt begins to interact exclusively with CS rather than DN. This salting-out phenomenon is evidenced by additional peaks in the CDC25 and CDC30 spectra compared to other electrolyte samples. Despite the salting-out effect, these samples exhibit high conductivity, likely due to the migration of salt ions through the channels within the dextran polymer, which is evident from the SEM analysis, thereby enhancing ionic conductivity.

### 3.3 Impedance study

**Fig. 5:**
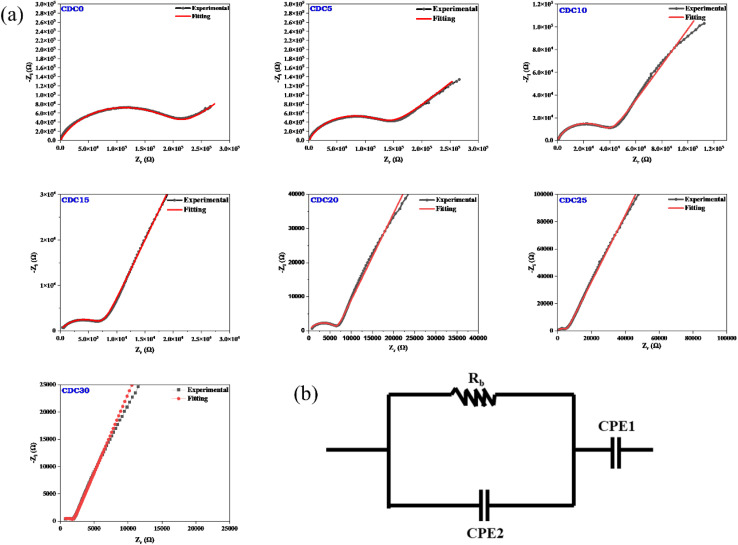
(a) Nyquist plot for pristine polymer and MgCl_2_ salt added electrolyte samples. (b) Model of the equivalent circuit.

Fig. 5(a) depicts the Nyquist plot for the CS : DN SBPE system at ambient temperature. The diagonal spike observed in the low-frequency area of the Nyquist plot is attributed to the ion-blocking effect at the interface between the electrode and the electrolyte. The constant phase element (CPE1) represents this blocking phenomenon. The interactions between the bulk resistance (*R*_b_) and bulk capacitance affect the system's overall impedance, as shown by the depressed semicircle. The bulk resistance is responsible for the ohmic losses in the system, while the bulk capacitance is responsible for the capacitive losses. The parallel combination of these two components results in the depressed semicircle shape.^49,50^ The bulk resistance reflects the resistance faced by the Mg^2+^ ions within the sample's bulk, while the bulk capacitance (CPE2) signifies the dipolar polarization of the polymer backbone chain.^51^ In polymer electrolyte systems bulk capacitance is associated with the dielectric of the polymer and capacitive losses are associated with energy dissipation in the presence of AC signal due to ion-conduction in the polymer matrix therefore, the highest conducting electrolyte system must exhibit the highest capacitive loss (dielectric loss) due to conduction of more number of free ions. A parallel combination is suggested because the process co-occurs. When an alternating current electrical field is applied, ions travel through the electrolyte membrane and are available at electrolyte/electrode interface. There was no charge transfer between the electrodes and the electrolyte because the electrodes were blocking electrodes. This meant that the system's impedance was only due to the resistance of the electrolyte and the double-layer capacitance.

**Fig. 6:**
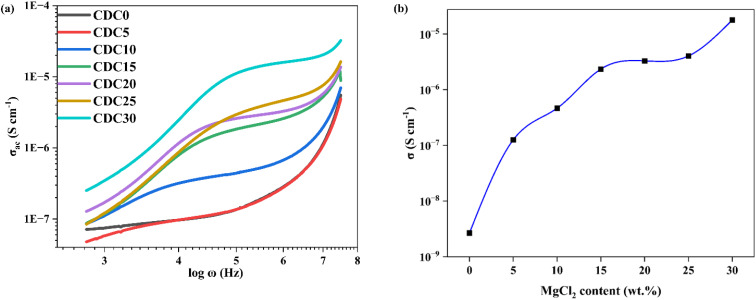
(a) Conductance spectra for the prepared SBPE membranes containing different MgCl_2_ salt content. (b) Influence of varying concentrations of MgCl_2_ salt on the ionic conductivity.

Consequently, it became feasible to independently ascertain the real and imaginary parts of the complex impedance across different frequencies. As the salt concentration rises, the bulk resistance of the SBPE system diminishes. The bulk resistance value is determined by fitting the data from the Nyquist plot to the parallel circuit model presented in Fig. 5(b). The parallel circuit model comprises a resistor (*R*_b_) and a capacitor (CPE2). The fitting process utilizes EIS spectrum analyzer software. The solid red line represents the fitted spectrum achieved by considering the equivalent circuit. Eqn (1) is a formula used to determine a sample's bulk ionic conductivity. The ions' ease of movement through a sample's bulk is gauged by its bulk ionic conductivity. The equation takes two inputs: the sample's *R*_b_ value and the thickness (*t*) of the sample.^52^Fig. 6(b) illustrates the variation in the bulk ionic conductivity of the sample with increasing salt concentration. The ionic conductivity depends on the salt incorporated into the polymer matrix and its degree of dissociation within the polymer. The polymer network's randomly oriented polar side groups facilitate ion movement within the sample bulk, reducing bulk resistance. This phenomenon occurs because the random orientation of polar side groups prevents the formation of large aggregates that could impede ion pathways. This indicates that the SBPEs are non-capacitive, which allows for more efficient energy storage by minimizing unwanted leakage and self-discharge, thereby preserving the battery's charge for more prolonged periods.^53^ At ambient temperature, the undoped blend system shows a conductivity of 1.03 × 10^−9^ S cm^−1^. Upon the incorporation of magnesium salt into the biopolymer blend matrix, the conductivity shows a consistent rise. The increase in ionic conductivity is due to the enhancement in the number of free ions available for conduction. Both magnesium and chloride ions are responsible for ionic conductivity. Ionic conductivity is related to charge density using the relation (4),

4 *σ* = *nqμ*

Among the various SBPEs, CDC30 showcases the highest conductivity, quantified at 1.79 × 10^−5^ S cm^−1^. This value closely corresponds to the 7.23 × 10^−6^ S cm^−1^ conductivity reported by Mustafa *et al.*^54^ for the addition of 30 wt% KOH. For high salt-added polymer electrolyte samples, *i.e.*, CDC25 and CDC30, ion clusters are formed, as is evident from the XRD results. Charge transfer becomes relatively faster with increased ionic clusters as shown below:M^+^X^−^M^+^ + X^−^M^+^ → M^+^X^−^ + M^+^X^−^M^+^

These activated cations jump would not only be limited to such small ionic clusters or ion pairs but also involve cation–biopolymer bond exchange. Since MgCl_2_ interacts more with CS than DN in higher concentrations of salt, these ion clusters move in the channels of DN polymer, leading to increased ionic conductivity, which is evident from the SEM analysis.

### 3.4 Conductance spectra analysis



5

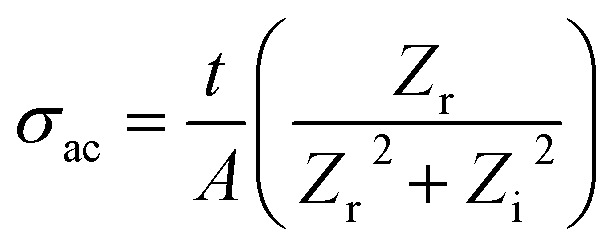


Fig. 6(a) depicts the changes in the ac conductivity spectra based on frequency for the SBPE 60CS/40DN/*X* MgCl_2_ (where *X* ranges from 0 to 30 wt% in increments of 5 wt%) under room temperature conditions. The ac conductivity spectra exhibit a clear relationship between salt concentration and the ionic conductivity of the polymer electrolyte.^55^ With increasing salt content, there is a corresponding rise in ionic conductivity. Within the observed frequency range, the conductivity's dependency on frequency can be categorized into three distinct phases: a low-frequency dispersion phase, an intermediate-frequency plateau region, and a high-frequency dispersion phase. The polymer electrolyte typically demonstrates low conductivity at low frequencies because ions have adequate time to accumulate at the electrode/electrolyte interface, forming a double-ion layer that opposes the applied electric field. The frequency-independent plateau observed in the ac conductivity spectra is associated with the dc conductivity (*σ*_dc_) of the SBPE samples. This plateau indicates that the sample's conductivity remains unaffected by changes in frequency, suggesting minimal frequency dependence on conductivity.^56^ At higher frequencies, the ions have more energy, which allows them to hop between different sites more easily. This results in an increase in conductivity.

### 3.5 Dielectric analysis

In the study of dielectric spectra, the electrolyte is sandwiched between the electrodes forming an interface at the electrode/electrolyte contact. During the dielectric polarization, the ions accumulate at the interface forming a double layer of capacitance at the interface and motions of the ions in the bulk have resulted in energy dissipation and thus dielectric loss.

6 ε*(*ω*) = *ε*′(*ω*) − j*ε*′′(*ω*)

**Fig. 7:**
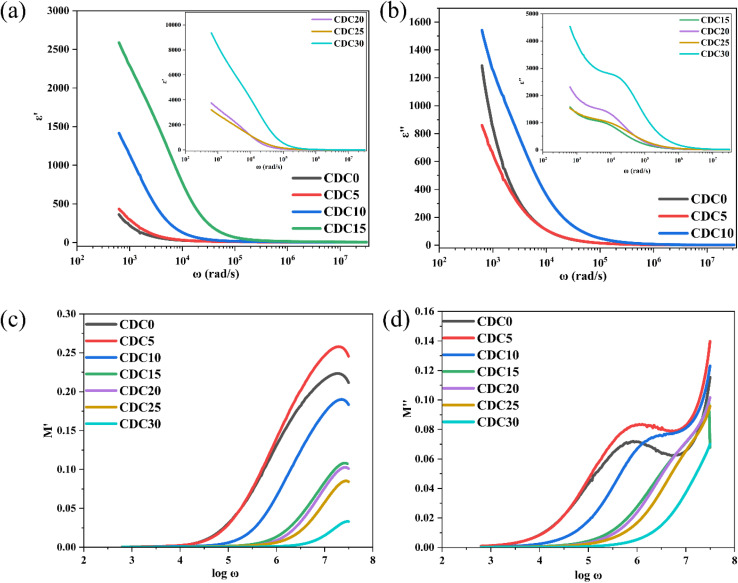
(a) Relationship between dielectric constant and angular frequency (*ω*) for all electrolyte samples. (b) Relationship between dielectric loss and angular frequency (*ω*) for all electrolyte samples. Frequency dependence of (c) *M*′(*ω*) and (d) *M*′′(*ω*) for the prepared samples.

The dielectric permittivity (ε*) is a complex quantity with real and imaginary components as given by eqn (6). The real component *ε*′ represents a material's capacity to store electric energy. The imaginary component, denoted as *ε*′′, indicates a material's capacity to release electrical energy as heat.^57^ At low frequencies, the value of *ε*′ decreases with increasing frequency. Conversely, at higher frequencies, the *ε*′ value levels off and stabilizes, as illustrated in Fig. 7(a). A rapid recurring electric field reversal causes the long tail at high frequencies.^58^ The abrupt rise in *ε*′ and *ε*′′ suggests the existence of electrode polarization and space charge effects, indicating a non-Debye type of relationship.^59^ With higher salt content, both the real and imaginary components of dielectric permittivity (*ε*′ and *ε*′′) consistently show an increasing trend across all polymer electrolyte samples at low frequencies. This suggests a rise in carrier concentration (the quantity of ions in the electrolyte) with increasing salt content.^60^ Inhibition of crystal growth and decreased ion–ion interaction results from a high dielectric constant, which also increases conductivity.^61^ The non-Debye characteristics of the prepared polymer electrolytes have been confirmed through dielectric analysis and modulus spectrum analysis, as presented in Section 3.6.^62^

### 3.6 Modulus spectrum analysis



7

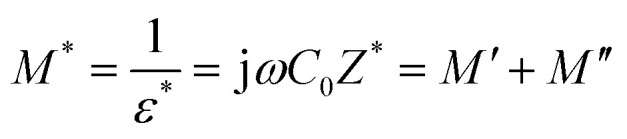



8

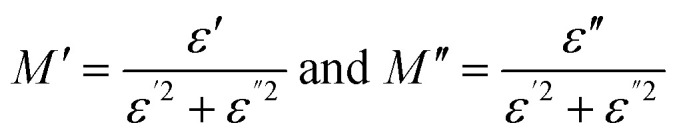

here, *ω* = 2π*f* represents the angular frequency, and *C*_0_ stands for the vacuum capacitance of the electrochemical cell.^63^

As was said in the section above, the other relaxation effect is suppressed at low frequencies because the space charge brought on by electrode polarization predominates. The data should be reported using modulus formalism to address this problem and minimize the impact of electrode polarization.^64^Fig. 7(c) and (d) show how *M*′ and *M*′′ change with frequency at room temperature. With increasing frequency, the values of *M*′ rise. Shukur *et al.*^65^ have observed similar results. A higher capacitance value of SBPE is indicated by the prolonged tail at low frequencies.^66^ The observed peaks in the plot of *M*′′ *vs.* log *ω* indicate dipolar activity, which aligns with the relaxation peaks depicted in Fig. S2.† The observed shift of the peak towards the higher frequency range is likely due to the ion hopping mechanism, associated with the microstructure modification brought about by ions in the electrolyte. This phenomenon arises as the sample's crystallinity diminishes in response to the rising concentration of salt, which is also evident from the XRD analysis.

### 3.7 Relaxation time

Fig. S2† illustrates the frequency dependence of the loss tangent (tan *δ*). The broad nature of the observed peak in the tan *δ* spectra can be attributed to the distribution of relaxation times. This implies the presence of a range of relaxation times within the system, and the peak in the tan *δ* spectra arises from the overlap of these various relaxation processes.^67^ A single relaxation peak has been consistently observed in all SBPEs, indicating that conduction relaxation predominates in these electrolytes. When subjected to an electric field, the energy dissipates as heat is gauged by tan *δ*. This loss tangent is defined as the ratio of the imaginary to real components of the complex permittivity. As illustrated in Fig. S2,† variations in MgCl_2_ concentration markedly affect the magnitude of conduction relaxation peaks in the loss tangent spectra.

Moreover, as MgCl_2_ content increases, the relaxation peaks gradually shift towards higher frequencies. This loss tangent spectra peak alteration may be ascribed to the expanding amorphous region within the solid polymer electrolyte. The conductivity relaxation time (*τ*) was obtained from loss tangent spectra using eqn (9).9
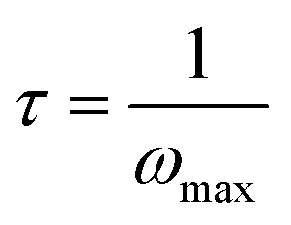
here, *ω*_max_ refers to the frequency at which the loss tangent peak reaches its maximum value. Fig. S2† depicts the peaks linked to translational ion dynamics, which correspond to the relaxation of mobile ion conductivity. The loss tangent peaks shift towards the high-frequency side, as the result of faster ion dynamics. The CDC30 sample exhibits the briefest relaxation time, measuring 9.62 × 10^−7^ s, alongside the highest ionic conductivity of 1.79 × 10^−5^ S cm^−1^.

### 3.8 Scanning electron microscopy (SEM)

**Fig. 8:**
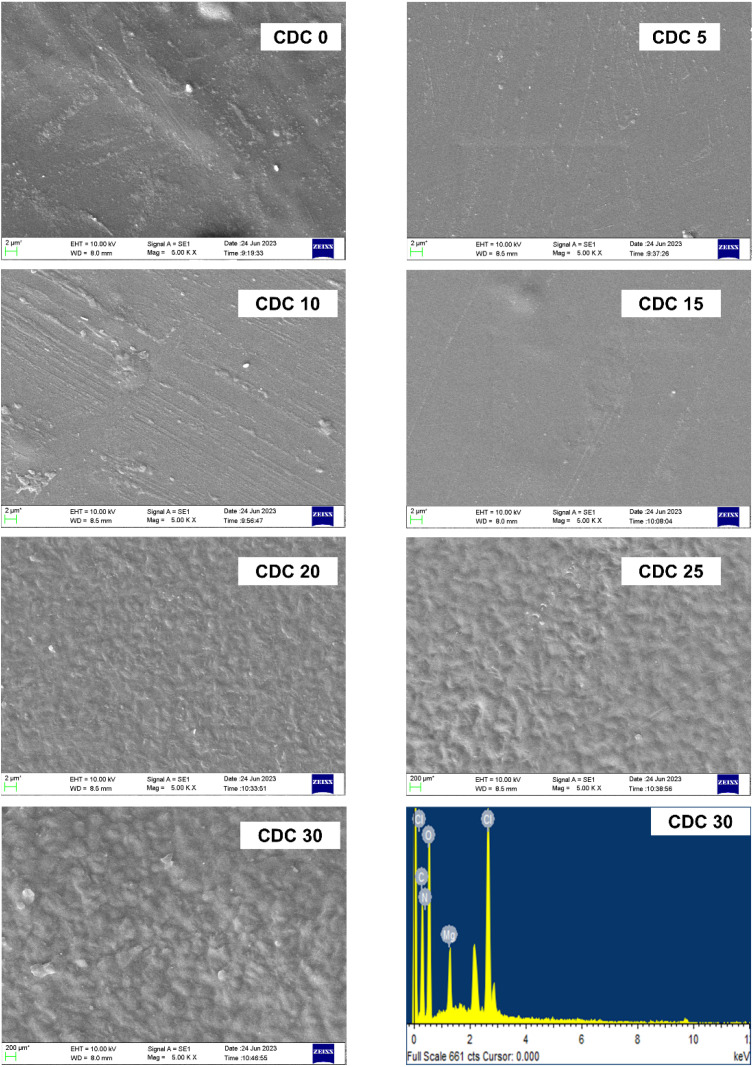
SEM micrographs of pure polyblend and blends doped with different wt% of magnesium chloride salt along with EDAX image (bottom right) of the highest conducting sample (CDC30).

Fig. 8 shows SEM images of the pure polymer blend and the blend containing different weight percentages of magnesium chloride salt. SEM images offer insights into the surface morphological characteristics of the SBPE samples. The surface morphology of the electrolyte films displayed a consistent and smooth structure, which was uniform across both the pure and doped blends. There were no cracks, and the agglomeration of the salt can be seen even at higher concentrations. These findings agree with those from XRD. A salting-out effect is evident from the XRD analysis for a higher salt concentration, *i.e.*, for CDC25 and CDC30 samples. Because of this salting-out effect, the salt interacts more with CS polymer than DN. So, the channels in the DN will be formed, which can be seen in the SEM micrographs. These channels are responsible for the movement of ion clusters, leading to high ionic conductivity, as discussed in the impedance study section (Section 3.3). Fig. 8 shows the Energy-Dispersive X-ray (EDAX) image of the polymer electrolyte sample boasting the highest conductivity (CDC30). The EDAX image confirms the presence of distinct elements (Mg, Cl, O, N, and C atoms) corresponding to the solid polymer blend electrolyte CS : DN : MgCl_2_. This suggests that the sample shows uniformity.

### 3.9 Atomic force microscopy

**Fig. 9:**
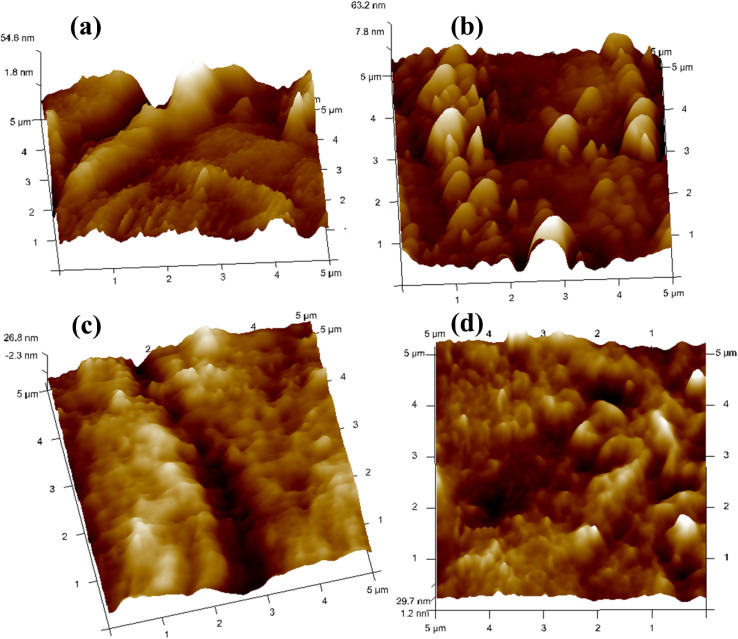
3D AFM images of (a) CDC0, (b) CDC10, (c) CDC20, and (d) CDC30 electrolytes.

The prepared polymer blend electrolytes CDC0, CDC10, CDC20, and CDC30 surface morphologies were investigated using AFM (Fig. 9), with the tip operated in tapping mode over a scanning area of 5 μm × 5 μm. It was observed that the pure blend CDC0 exhibited a root mean square (RMS) roughness of 34.7 nm. The roughness of CDC10 decreased to 14.9 nm, indicating a smoother morphology. After the incorporation of 10 wt% of MgCl_2_, the surface roughness is reduced, leading to a smoother surface and diminished crystallinity of the blended polymer electrolyte. A dependency of the roughness of the electrolyte surface on salt concentration has been observed in this study. The smoother surface will reduce the electrode–electrolyte interfacial resistance hence making it a best candidate for energy storage device applications. Similarly, the roughness values for CDC20 and CDC30 were 8.81 nm and 8.24 nm, respectively. Improving surface uniformity could potentially enhance electrode–electrolyte contact, thereby improving device performance. Regarding transport analysis, the parameter *p*_2_ signifies the deviation of the spike from ideal behaviour (*p*_2_ = 1). A value of *p*_2_ closer to unity suggests smoother sample characteristics.^68^ The *p*_2_ value for the CDC0 sample was 0.740, while for CDC30, it was 0.808, indicating that CDC30 is smoother compared to CDC0. This observation is consistent with SEM and XRD findings.

### 3.10 Thermal stability

Fig. S3† shows the TGA thermograms of the prepared SBPE films. The SBPE thermal stability was evaluated by subjecting it to various temperatures and analysing the mass loss. Thermal stability is essential for polymer electrolytes to ensure their performance. The thermal profiles of both the pure blend and the blend infused with MgCl_2_ show that the pure blend encounters an initial mass decrease of 20% at 210 °C, attributed to the evaporation of entrapped solvents. The pure blend film maintains its stability until it reaches a temperature of 230 °C. CDC30 exhibits stability up to 190 °C, retaining 76% of its mass. The onset of the second degradation occurs at 190 °C. All materials are stable up to 190 °C with an average mass loss of 24%. Considering that electrochemical energy storage devices usually function at temperatures significantly lower than 100 °C, our samples meet the criteria for thermal stability. The findings indicated that the pure blend remains stable until 230 °C, whereas the film with the highest conductivity (CDC30) maintains stability up to 190 °C. All samples with salt are stable up to 190 °C, which meets the thermal stability criterion for electrochemical energy storage devices.

### 3.11 *I*–*V* characterization

**Fig. 10:**
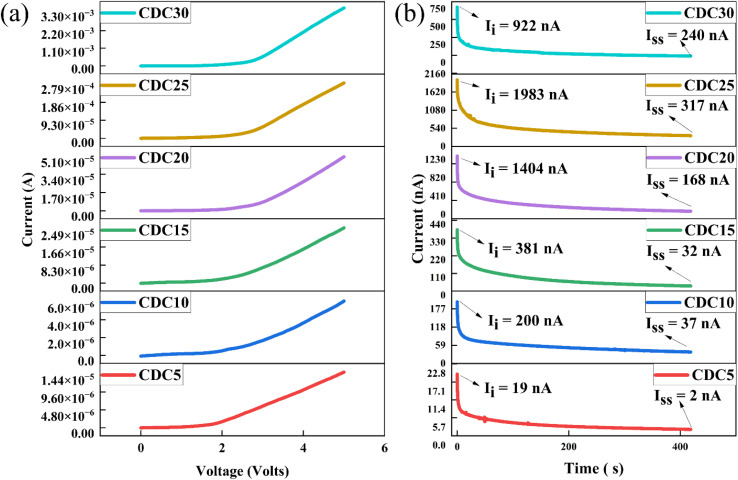
(a) A graph showing current as a function of voltage for the SS|[CS : DN] : MgCl_2_|SS cell. (b) Polarization plot of the prepared polymer electrolyte samples.

The electrochemical stability window (ESW) is significant when producing electrochemical devices. It signifies the voltage range in which the polymer electrolyte remains unaffected by oxidation or reduction reactions. This study utilized a Keithley source meter to ascertain the ESW of all the prepared SBPEs experimentally. The results indicated minimal current observed below 2.0 V, indicating the absence of any electrochemical reactions, as depicted in Fig. 10(a). Upon crossing the 2.0 V threshold, there was a gradual rise in the current. The estimation of the ESW for the CDC30 sample was achieved by extending the linear current from higher voltages to intersect the *x*-axis. The computed ESW was 2.66 V, consistent with values documented in prior literature.^69^ Beyond this voltage, there was a notable and rapid rise in the current, indicating the electrolyte breakdown occurring at the inert electrode surfaces. S. B. Aziz *et al.*^70^ also determined that the ESW for CS : DN : NH_4_I electrolyte and was 1.8 V.

### 3.12 Transference number analysis

Fig. 10(b) shows the polarization plot of the SBPEs. The total conductivity of an SBPE is due to the combined contributions of ions and electrons.

Nonetheless, in the context of batteries and EDLC applications, ions, especially cations, are expected to be the primary charge carriers, while electron conductivity remains significantly lower (*t*_ion_ ≫ *t*_ele_).^71^ This phenomenon arises from the stainless steel (SS) electrodes, renowned for their ability to impede ion flow. The ionic transference number (*t*_ion_) is determined for all polymer electrolyte samples using eqn (2). For the polymer electrolyte film with the highest ionic conductivity (CDC30), *t*_ion_ is calculated to be 0.73, indicating that ions predominantly act as the primary charge carriers within this highly conductive sample. This value, approximately 0.73, arises from the combined influence of the Mg salt's cation and anion. The limited electronic conductivity displayed by the prepared electrolytes confirms their appropriateness for use as separators and electrolytes in battery applications.^72^

### 3.13 Cyclic voltammetry (CV) of the prepared EDLC device

**Fig. 11:**
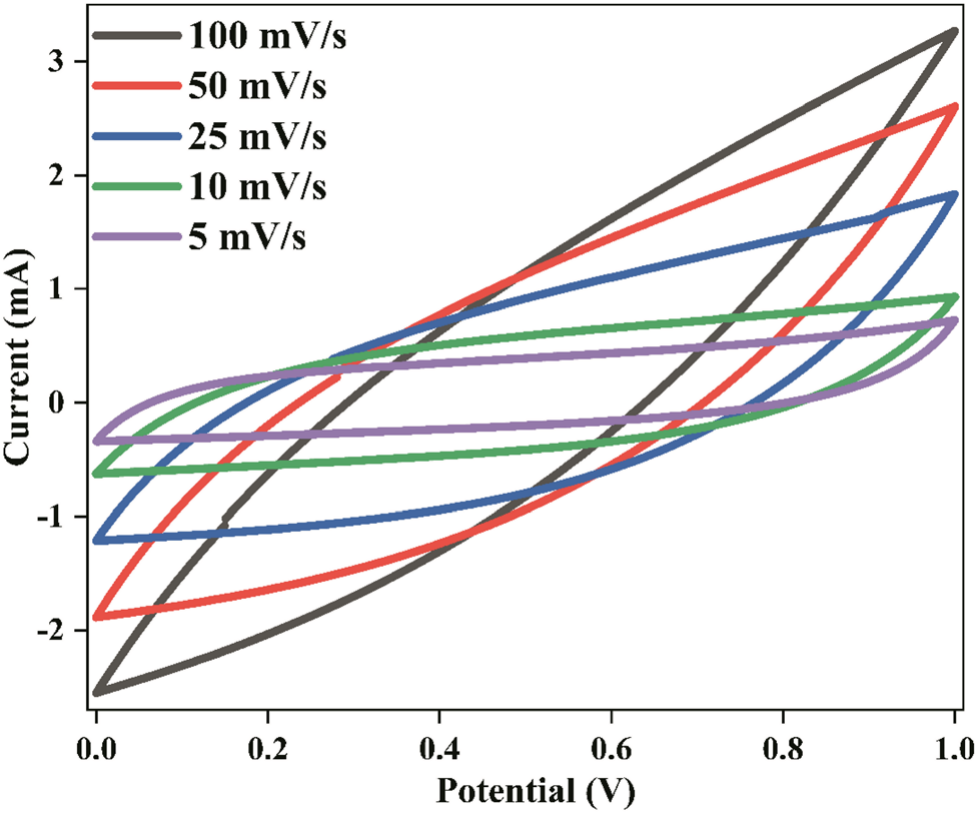
Cyclic voltammogram of EDLC recorded at various scan rates.

CV was conducted during the EDLC characterization process, as shown in Fig. 11. The impact of scan rate on the shape of the CV consistently exhibits a leaf shape across all scan rate values. While an ideal capacitor displays a perfect rectangular shape in the CV response, real capacitors are influenced by various factors, such as electrode porosity and internal resistance, affecting the relationship between current and voltage.^73^ The absence of oxidation/reduction peaks in the recorded CV response indicates a non-faradaic energy storage mechanism in the EDLC, where adsorption and desorption processes of cations and anions occur at the negative and positive electrodes, respectively. The specific capacitance (*C*_cyc_) was determined from the CV using the provided eqn (10):10
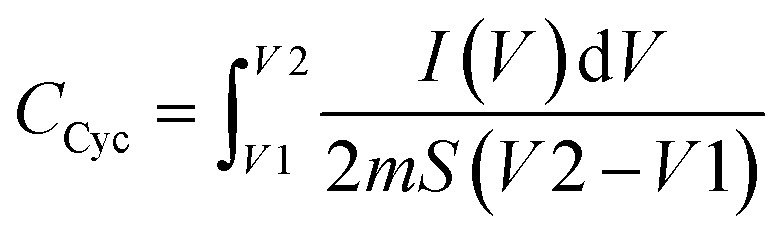


The area of the CV profile, denoted by ∫*I*(*V*)d*V*, was computed using OriginPro 2021 software. This study conducted the CV within the voltage range from *V*1 (0 V) to *V*2 (1 V), with ‘*m*’ representing the mass of active material used and ‘*S*’ representing the scan rate.

The calculated values of *C*_cyc_ are presented in Table 2. Higher *C*_cyc_ values are observed at lower scan rates, whereas this trend is not observed at higher scan rates. In this study, ions establish a stable double-layer charge at the interfacial region between the AC electrode surfaces during low scan rates. Under these conditions, a significant capacitance value is achieved. An almost perfect plateau region is observed at low scan rates, indicating that free ions migrate relatively constantly, accumulating ions at the electrode–electrolyte interface with minimal ohmic resistance. Notably, a thick diffuse layer forms at low scan rates at the interface region between the electrolyte and the electrode. Conversely, a thin, diffuse layer at high scan rates promotes faster ionic conduction, which hampers the desired polarization process formation.^74^Table 3 compares the findings of this study and those of previously reported studies on EDLCs utilizing carbon electrode materials, focusing on the specific capacitance values obtained from CV responses.

**Table 2:** Calculated specific capacitance from the CV plot at different scan rates

Scan rate (mV s^−1^)	*C*_cyc_ (F g^−1^)
100	5
50	10
25	18
10	27
5	36

**Table 3:** Comparison between the attained specific capacitance in the present investigation and those reported in previous research that employed cyclic voltammetry

System	*C*_cyc_ (F g^−1^)	Scan rate (mV s^−1^)	References
PVA + CS + NaBr + Glycerol + CaTiO_3_	28.65	10	75
MC + NaSCN + Glycerol	55.22	10	76
CS + POZ + NaBr + Glycerol	22.63	20	77
MC + NH_4_NO_3_ + [BMIM]BF_4_	124.05	01	78
CS + PVA + NaOAc + Glycerol	38	20	74
PS + CS + LiClO_4_ + Glycerol	38.68	20	73
DN + CS + MgCl_2_	36	05	This work

### 3.14 Electrochemical impedance spectroscopy (EIS)

**Fig. 12:**
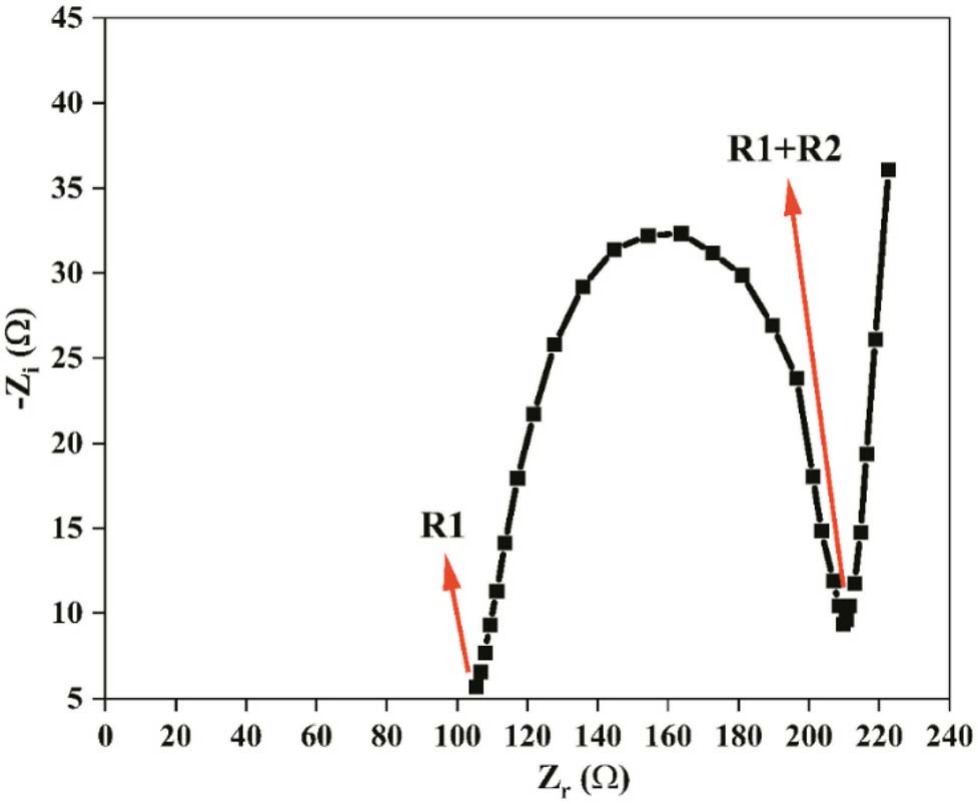
Nyquist plot of EDLC.

Fig. 12 depicts the Nyquist impedance plot of the assembled EDLC within the frequency range from 100 Hz to 1 MHz at room temperature. At a high-frequency range, the impedance plot does not intersect the origin point, indicating the presence of resistance, referred to as the capacitor cell's bulk resistance (*R*1). The bulk resistance comprises the resistance of the polymer electrolyte, connector series resistance, internal resistance of the electrode for ion diffusion, and ohmic loss of the cell.^79^

Following the *R*1, a depressed semicircle is observed in the high-frequency range. This semicircle comprises a capacitor and a resistor connected in parallel. The capacitor represents the capacitance of the double layer (*C*_dl_), which arises from the formation of an electrical double layer at the electrode–electrolyte interface due to ion accumulation between the electrode and electrolyte when ions diffuse in the electrolyte and adsorb into the porous carbon electrode. The resistance, known as charge transfer resistance (*R*2), is a bulk behaviour of the electrode–electrolyte interface. This resistance represents the minimum energy required to form the electrical double layer at the electrode–electrolyte boundary. Mobile charge carriers must overcome this resistance to diffuse in the electrolyte and accumulate in the pores of the carbon electrode. In this study, the values of *R*1 and *R*2 for the fabricated EDLC are 105 Ω each.

A linear steep rising curve is observed at the low-frequency end, indicating the presence of Warburg impedance (*W*_o_), which is associated with ion diffusion into the porous carbon. This linearly rising pattern signifies ion adsorption at the surface between the carbon-based electrode and polymer electrolyte, forming the electrical double layer. This pattern further confirms the capacitive behaviour of the EDLC, as represented by the constant phase element (CPE) of the EDLC.

### 3.15 Cyclic voltammogram (CV) studies of battery setup

**Fig. 13:**
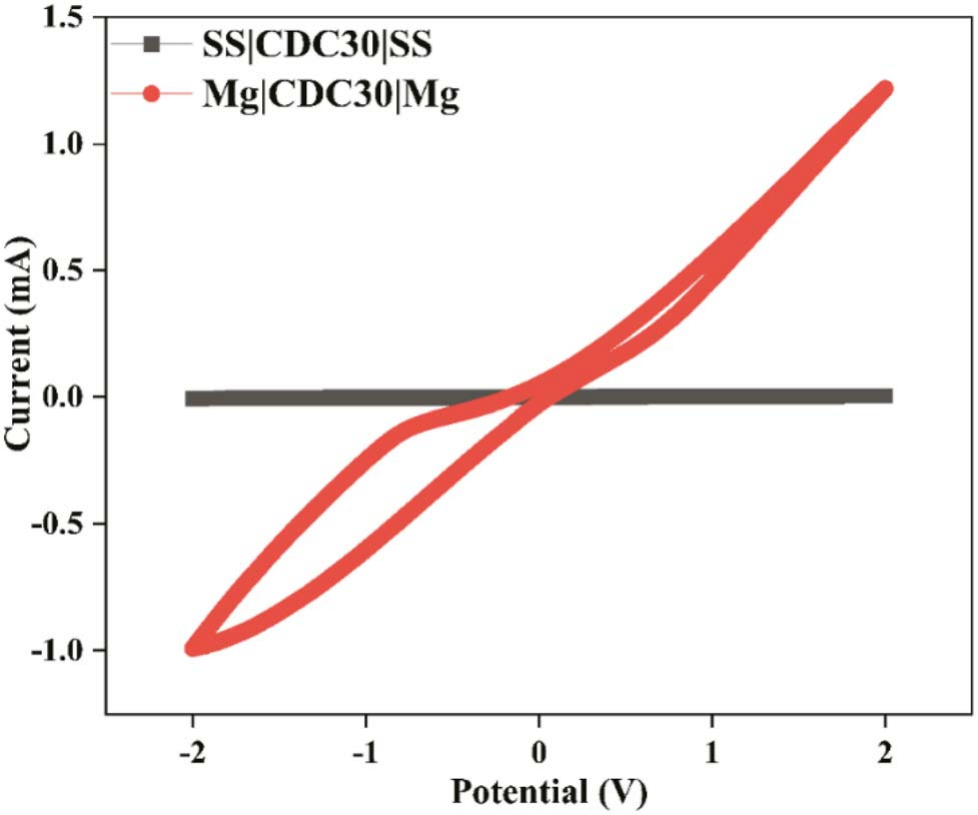
CVs of the SBPE sample with the highest ion conductivity.

Two symmetrical cells were used to test how well magnesium ions can be plated and stripped in an SBPE. Fig. 13 depicts the CV of:

Cell A: SS/CDC30/SS.

Cell B: Mg/CDC30/Mg.

The potential was varied from −2.0 V to +2.0 V at a speed of 100 mV s^−1^.^80^ The first cell's current window is minuscule compared to the second one. Moreover, the first cell has no clear cathodic and anodic peaks, whereas the second exhibits minor redox peaks. This demonstrates that SBPE films conduct Mg^2+^ ions reversibly and have the required electrochemical stability for the magnesium ion battery application.Mg^2+^ + 2e^−^ ↔ Mg^0^

### 3.16 Analyses of Mg ion primary batteries with various cathode materials

**Fig. 14:**
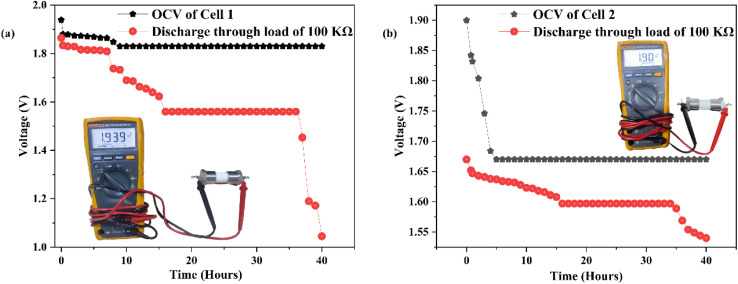
(a) The open circuit voltage (OCV) of cell 1 (photo of cell 1 shown in the inset). (b) The open circuit voltage (OCV) of cell 2 (photo of cell 2 shown in the inset).

Fig. 14(a) and (b) depicts the assembly of a primary battery that conducts magnesium ions. This battery configuration includes the CDC30 polymer electrolyte sandwiched between a magnesium metal pellet (anode) and two distinct cathode materials. In the context of all-solid-state batteries, the selection of the anode material is influenced by the mobile species present in the electrolyte, leading to the choice of a magnesium metal pellet as the anode. Two different cathode materials have been employed to explore the impact of electrode material on SBPE performance.

The incorporation of graphite enhances the electronic conductivity of the cathode materials. In the second cell, iodine is utilized as an “active cathode” material. Adding the SBPE with the electrode material increases the surface area for interfacial interaction within the cathode assembly. Consequently, the interfacial resistance between the electrode and the electrolyte decreases, enhancing battery performance.

Table 2S† presents a summary of the cell properties. In the first cell, the initial open circuit voltage started at 1.94 V, then declined to 1.83 V, and maintained stability for 40 hours. During these 40 hours, the voltage decreased further to 1.05 V upon applying a load of 100 kΩ. Cell 2's initial open circuit voltage began at 1.90 V, reduced to 1.67 V, and remained consistent for 40 hours. When a 100 kΩ load was connected, the voltage decreased from 1.67 V to 1.54 V over 40 hours. The findings of this current research align with those from prior studies, as depicted in Table 4. This study highlights the impressive electrochemical capabilities of the biopolymer blend electrolyte, which not only provides cost-effectiveness but also showcases renewable and environmentally friendly attributes.

**Table 4:** Comparison between the attained OCV in the present investigation and those reported in previous research that employed a magnesium-based anode

Electrolyte	Cathode	OCV	References
PVA + PVP + Mg(NO_3_)_2_	I_2_ + C + electrolyte	1.85	81
PVA + PAN + Mg(ClO_4_)_2_	MnO_2_	2.06	82
PVA + PEG + Mg(CH_3_COO)_2_	I_2_ + C + electrolyte	1.84	83
PVA + PAN + Mg(NO_3_)_2_	MnO_2_ + C	2.02	84
CS + MC + MgCl_2_	MnO_2_ + C + electrolyte	2.15	27
CA + Mg(NO_3_)_2_	MnO_2_ + C + electrolyte	2.1	85
DN + CS + MgCl_2_	MnO_2_ + C + electrolyte	1.94	Present work
DN + CS + MgCl_2_	I_2_ + C + electrolyte	1.90	Present work

### 3.17 Test for flammability

**Fig. 15:**
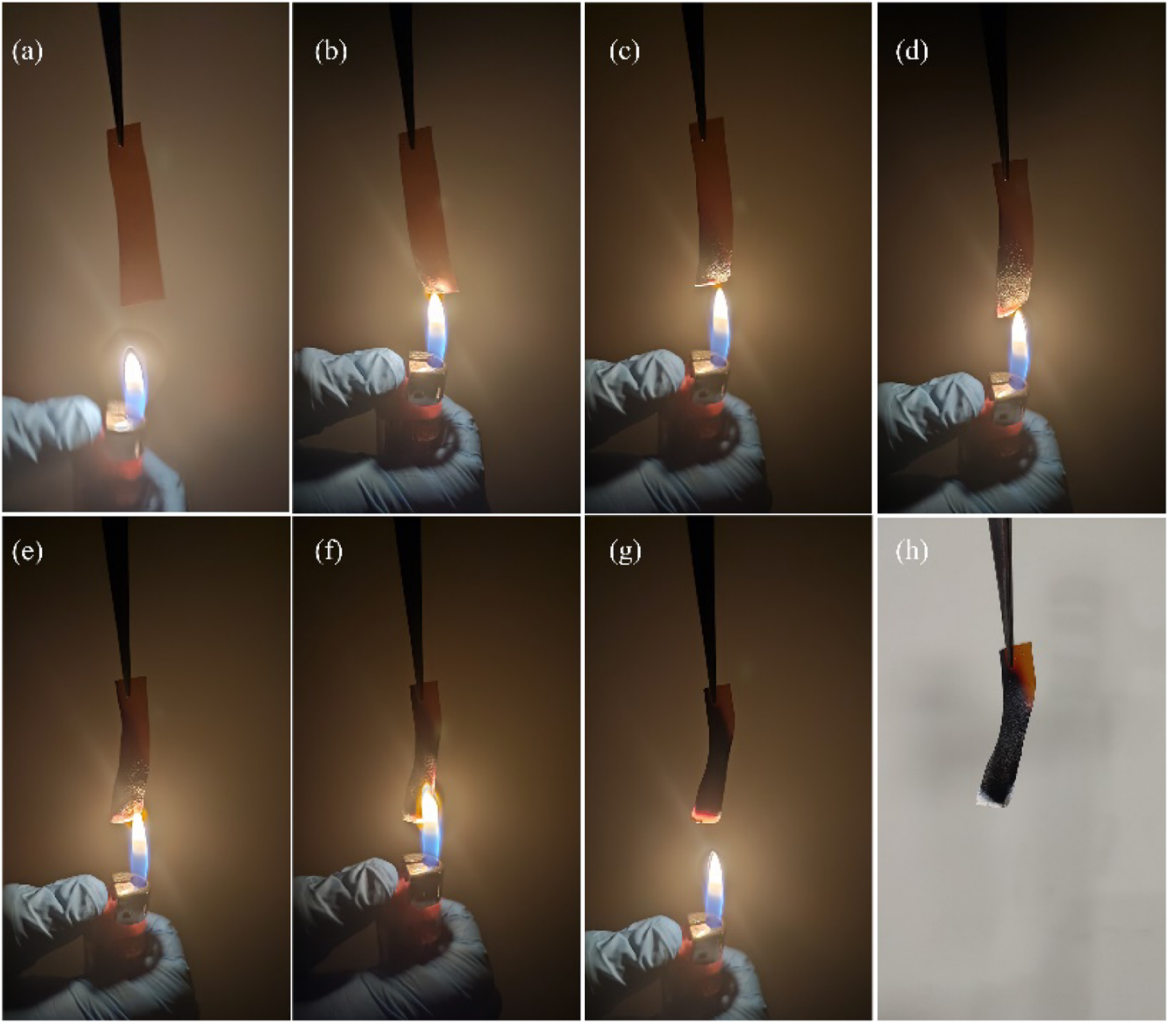
(a)–(h) Various phases of the flame test procedure for CDC30.

The flammability evaluation of CDC30 was carried out using a flame test (Fig. 15). When subjected to a flame, the electrolyte sample underwent combustion, forming ashes without any visible flames. This indicates that the decomposition of the polymer electrolyte occurs without producing flammable substances.^86^

## 4. Conclusions

Solid blend polymer electrolyte (SBPE) films containing dextran, chitosan, and magnesium chloride salt are produced using the solution casting method. FTIR analysis outcomes reveal the establishment of hydrogen bonds between the two polymers and cohesive compatibility between the polymers and magnesium salt. According to XRD analysis, CDC30, which boasts the highest conductivity, exhibits the least crystallinity among these systems. However, its stability diminishes when temperatures exceed 190 °C. The CDC30 sample demonstrates an ionic conductivity of 10^−5^ S cm^−1^ and a total ionic transference number of 0.73. Dielectric analysis outcomes reveal that both *ε*′ and *ε*′′ values decrease with increasing frequency, suggesting non-Debye behavior across all studied samples. The *I*–*V* analysis highlights the suitability of the most conductive electrolyte for EDLC and battery-related applications.

Additionally, it is demonstrated that the electrolyte system displays significant capacitive behavior. The impact of scan rate on specific capacitance is elucidated, with lower scan rates leading to increased specific capacitance, while higher scan rates exhibit the opposite effect. The most conductive polymer electrolyte membrane, comprising 60 wt% CS, 40 wt% DN, and 30 wt% MgCl_2_, is employed as an electrolyte for constructing a primary magnesium battery, yielding an open circuit voltage (OCV) of approximately 1.9 V. These results collectively signify that the developed blend polymer electrolyte possesses favourable structural and electrochemical attributes, rendering it suitable as a combined separator and electrolyte membrane for magnesium-ion batteries and EDLC device applications. Future research could focus on refining magnesium and magnesium-based composite electrodes alongside the SBPE for secondary battery applications.

## Data availability

The data that support the findings of this study are available from the corresponding author upon reasonable request.

## Author contributions

Pradeep Nayak: conceptualization, methodology, data curation, formal analysis, investigation, writing-original draft. Ismayil: methodology, validation, supervision, writing – review & editing. Y. N. Sudhakar: visualization, resources, data curation, investigation. Supriya K. Shetty: formal analysis, investigation.

## Conflicts of interest

The authors declare that they have no known competing financial interests or personal relationships that could have appeared to influence the work reported in this paper.

## Supplementary Material

RA-014-D4RA06365A-s001
