# Supplementary material for: Charging toward sustainability: MgCl2 doped chitosan–dextran polyblend electrolytes for energy storage device applications
Source: RSC Adv. 2024 Nov 19;14(50):37045–61. doi: 10.1039/d4ra06365a (PMC11575477; doi:10.1039/d4ra06365a)
Supplement: RA-014-D4RA06365A-s001 [file RA-014-D4RA06365A-s001.pdf]

Supplementary Information (SI) for RSC Advances.
This journal is © The Royal Society of Chemistry 2024

Supporting information

Charging Toward Sustainability: MgCl2 doped Chitosan-Dextran Polyblend Electrolytes for Energy Storage Device Applications

Pradeep Nayak[a], Ismayil[a*], Y. N. Sudhakar[b], Supriya K. Shetty[a]

[a]Department of Physics, Manipal Institute of Technology, Manipal Academy of Higher Education, Manipal 576104, Karnataka, India

[b]Department of Chemistry, Manipal Institute of Technology, Manipal Academy of Higher Education, Manipal 576104, Karnataka, India

*Corresponding Author e-mail: ismayil.mit@manipal.edu, ismayil.486@gmail.com

Tel: +91 98454 97546

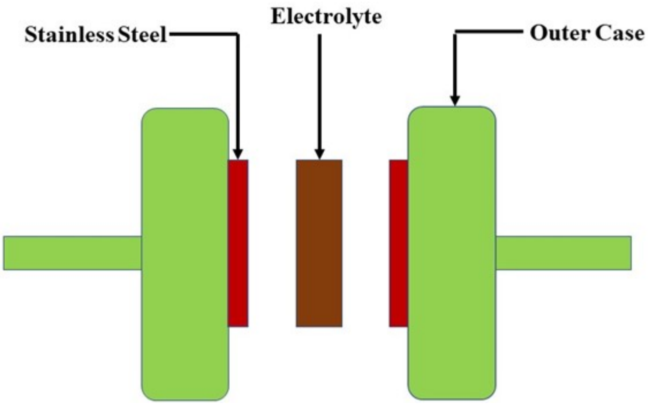

Figure S1. Standard cell arrangement utilized for conducting I-V and I-T investigations

Table 1S: FTIR peak positions for all the prepared SBPE.

FTIR peak Assignment	Wavenumber (cm^{-1})						
	CDC0	CDC5	CDC10	CDC15	CDC20	CDC25	CDC30
-OH Stretching	3277	3267	3266	3257	3247	3266	3266
C-H Stretching	2920	2919	2918	2917	2918	2917	2917
-NH Bending	1551	1547	1549	1550	1549	1548	1530
C-H Bending	1410	1410	1412	1412	1413	1415	1414
C-O Bands	1012	1010	1008	1009	1011	1013	1012

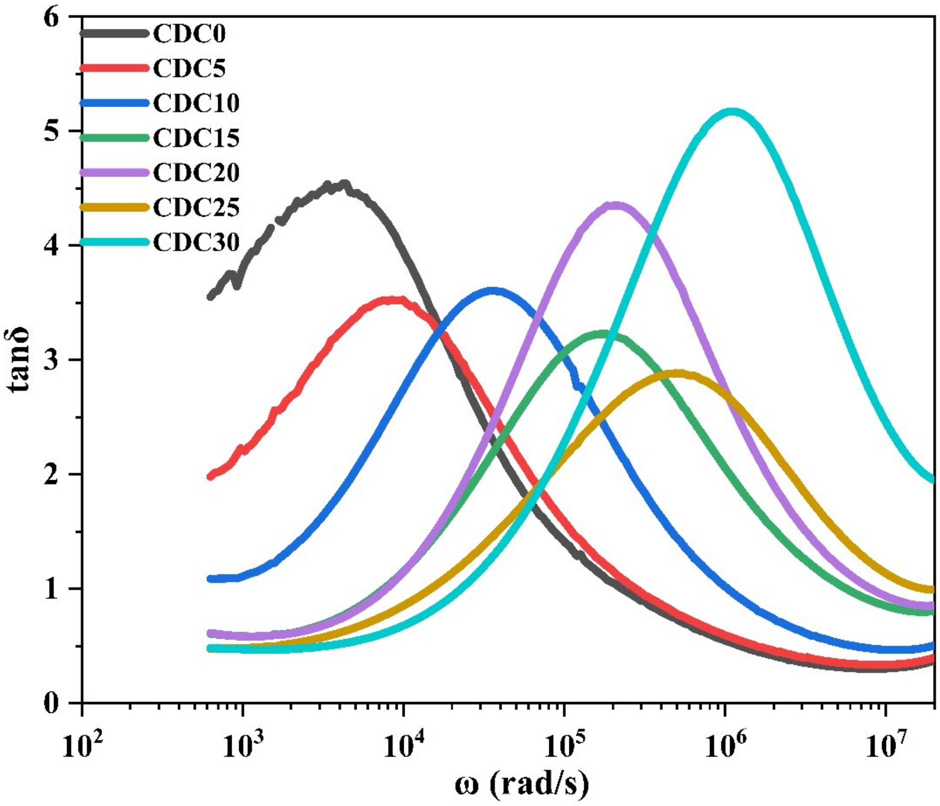

Figure S2. Tangent delta plot for the prepared polymer electrolyte samples.

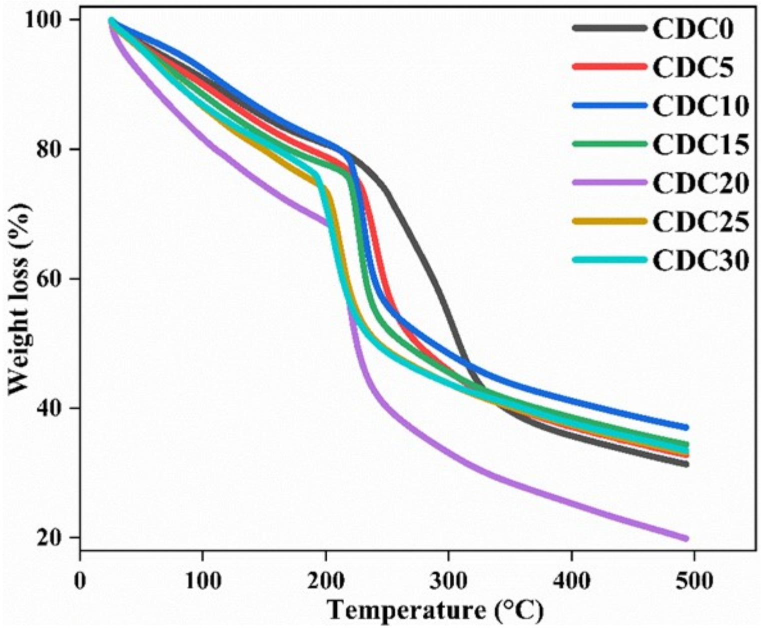

Figure S3. TG thermogram for CS+MC with different concentrations of $MgCl_2$

Table 2S: Parameters of the cell

**Cell parameters**	**Cell 1**	**Cell 2**
Cell area (cm^2)	1.23	1.23
Weight of the cell (g)	1.11	1.02
Effective diameter of the cell (cm)	1.30	1.30
Thickness of the cell(cm)	0.44	0.44
Open circuit voltage (V)	1.94	1.90
Current drawn (mA)	1.6	1.9
Current density ($mAcm^{-2}$)	1.30	1.54
Discharge time plateau region (h)	20	19
Discharge capacity (mAh^{-1})	0.08	0.1
Energy density ($Whkg^{-1}$)	56	67
Power density (Wkg^{-1})	3	4
